# Sleep loss impairs cognitive performance and alters song output in Australian magpies

**DOI:** 10.1038/s41598-022-10162-7

**Published:** 2022-04-22

**Authors:** Robin D. Johnsson, Farley Connelly, Juliane Gaviraghi Mussoi, Alexei L. Vyssotski, Kristal E. Cain, Timothy C. Roth, John A. Lesku

**Affiliations:** 1 School of Agriculture, Biomedicine and Environment, La Trobe University, Melbourne, Australia; 2 School of BioSciences, The University of Melbourne, Melbourne, Australia; 3 School of Biological Sciences, The University of Auckland, Auckland, New Zealand; 4 University of Zurich/ETH Zurich, Zurich, Switzerland; 5 Department of Psychology, Franklin and Marshall College, Lancaster, USA

**Keywords:** Neuroscience, Psychology, Zoology

## Abstract

Sleep maintains optimal brain functioning to facilitate behavioural flexibility while awake. Owing to a historical bias towards research on mammals, we know comparatively little about the role of sleep in facilitating the cognitive abilities of birds. We investigated how sleep deprivation over the full-night (12 h) or half-night (6 h) affects cognitive performance in adult Australian magpies (*Cracticus tibicen*), relative to that after a night of undisturbed sleep. Each condition was preceded and followed by a baseline and recovery night of sleep, respectively. Prior to each treatment, birds were trained on an associative learning task; on the day after experimental treatment (recovery day), birds were tested on a reversal learning task. To glean whether sleep loss affected song output, we also conducted impromptu song recordings for three days. Ultimately, sleep-deprived magpies were slower to attempt the reversal learning task, less likely to perform and complete the task, and those that did the test performed worse than better-rested birds. We also found that sleep-deprived magpies sang longer yet fewer songs, shifted crepuscular singing to mid-day, and during the post-recovery day, song frequency bandwidth narrowed. These results collectively indicate that sleep loss impairs motivation and cognitive performance, and alters song output, in a social adult songbird.

## Introduction

Sleep is important for optimizing waking performance of many animals. In awake animals, new memory traces are acquired and encoded in the brain. During subsequent sleep, newly formed memories are stabilized and enhanced via a process of memory consolidation^1,2^. Obtaining an insufficient amount of sleep negatively affects these neurological processes with consequences for subsequent performance^3–7^. Indeed, sleep loss impairs cognition, broadly defined to include attention, motivation, visual-motor coordination, emotional stability, communication, short- and long-term memory, and executive functions related to decision making^8–14^.

Most studies related to sleep-dependent cognition have been done on humans and a few other mammals; as a result, relatively little is known about the importance of sleep for cognitive processes in other taxa, including birds. From an evolutionary perspective, birds are particularly interesting because they are the only group of (non-mammalian) animals to show unequivocal mammal-like sleep states of alternating non-rapid eye movement (NREM) and REM sleep^15,16^. These sleep states appear to have evolved independently in each lineage through convergent evolution, although more study is needed on ectothermic vertebrates and invertebrates^17^. Interestingly, advanced cognitive abilities are also shared between birds and mammals, such as causal reasoning, flexibility, imagination, prospection, and tool use^18,19^. It is intriguing to speculate that the presence of advanced cognitive ability and unequivocal NREM and REM sleep in birds and mammals are inter-related^20^. In this way, the study of avian sleep-dependent cognition provides a unique opportunity to explore the evolution and cognitive function of sleep.

Of the little research conducted, sleep has been found to play a role in learning and memory for some birds, such as filial imprinting in chicks (*Gallus gallus domesticus*). Memory for imprinting is rapidly formed and stored in the brain^21,22^. The proportion of neurons associated with the imprinting stimulus increases after sleep, indicating the importance of sleep for encoding and consolidating imprinting memories^23,24^. Furthermore, chickens engage in more REM sleep^25^ and have greater 5–6 Hz NREM sleep brain activity^24^ after imprinting, however the significance of these findings remain unclear. In addition to imprinting, some research suggests a relationship between spatial memory and sleep. Chickens sleep more after learning a spatial discrimination task^26,27^, and Indian house crows (*Corvus splendens*) that displayed nocturnal restlessness from exposure to light at night, showed impaired spatial and visual memory during the day^28,29^. Conversely, migratory white-crowned sparrows (*Zonotrichia leucophrys gambelii*), which sleep two-thirds less at night relative to the same birds when in a non-migratory state, showed no impairment on an operant task, challenging a role for sleep in maintaining cognitive function in these birds^30^.

Behavioural flexibility (also known as cognitive flexibility), is an ecologically relevant cognitive domain that has been studied in birds, but never in relation to sleep. In studies of behavioural flexibility, it is common to measure learning in problem-solving situations, which is often quantified as the capacity of the animal to learn the reversal of a task on which they have been well trained^31^. Reversal learning tasks measure how quickly and successfully animals adapt to the change of reinforcement contingencies, and has been widely used in testing avian cognition^32–35^. Moreover, reversal learning tasks may reflect inhibitory control because they highlight the capacity to suppress and withhold a learned reward-winning behaviour^31,36^, although in great tits (*Parus major*) reversal learning did not correlate with inhibitory control^37^. The behavioural flexibility that characterizes reversal learning may be especially advantageous in complex and stochastic environments^33,38^.

Other cognitive domains, such as the learning of song in young birds, have been explored in relation to sleep. Songbirds communicate through singing^39^; their songs develop early in life by imitation and auditory feedback. By the time of sexual maturity, the songs of many passerines crystallize^40–42^. The ability of young songbirds, such as juvenile zebra finches (*Taeniopygia guttata*), to ultimately mimic adult song is a sleep-dependent process^43,44^. The involvement of sleep in song learning is not limited to juveniles. Adult starlings (*Sturnus vulgaris*) learn to discriminate between different songs and make a decision on which songs are relevant. Starlings are better at discriminating between novel conspecific song segments after periods of sleep^45^. Furthermore, starlings kept awake between training and testing had an impaired ability to distinguish between an interfering song segment and original songs^46^. Collectively, these studies reveal the importance of sleep in song learning in young songbirds, and song discrimination in adult songbirds. Nevertheless, what remains unclear is whether song output per se is dependent on the amount of prior sleep in adult birds.

Birdsong can encode information about the singer, such as condition and intent^39,47^. Individuals which produce physically demanding songs, such as trills (rapidly repeating song elements with high frequency bandwidth) receive stronger responses from both rivals and mates, and increased fitness^48–52^. Furthermore, complex song parameters including repertoire size, syntactical organization, stereotypy, and melody-like performance are especially important for more versatile singers. Male sedge warblers (*Acrocephalus schoenobaenus*) with a larger song repetoire pair with a female earlier^53^; female great reed warblers (*Acrocephalus arundinaceus*) respond more to males with complex songs and large repertoire size compared to other males^54^. In addition to the structural information within the songs, the timing and duration of singing activity is also an important signal^39^. To our knowledge, no study has examined how sleep might affect the song structure and song rate in passerines.

In this study, we investigated whether cognitive function is dependent on the amount of prior sleep in a songbird, the Australian magpie (*Cracticus tibicen,* also known as *Gymnorhina tibicen*). Australian magpies are known for their social nature, advanced cognitive abilities^55,56^, and complex vocal behaviours^55,57^. To see whether sleep loss affected cognitive performance, we tested adult Australian magpies on a reversal learning task, which measures behavioural flexibility, after full-night and half-night sleep deprivation. Also, we took the opportunity to serendipitously record their songs after full-night sleep deprivation. Magpie song (Supplementary Audio S1; Figure S1) serves several ecologically-relevant functions, including territorial and nest defence, food calling, and pair bonding, and is performed by the adults of both sexes^55,58^. We predicted the magpies’ performance on the reversal learning task would worsen, and birds would sing less after a night of extended wakefulness.

## Methods

### Animals and housing

In January 2019, 12 wild Australian magpies of the Victorian subspecies (*Cracticus tibicen tyrannica*; equally sexed) were caught in a walk-in trap baited with grated cheese. All magpies were non-breeding and non-paired adults without a territory (age and sex based on plumage; Connelly pers obs)^59^. All points of capture were in urban areas and parks in the City of Melbourne. Each bird was banded with a metal band and a numbered plastic leg band for individual identification. Immediately after banding, each bird was transported to the animal facility at La Trobe University, Melbourne. The magpies were housed individually in aviaries (1.8 m high × 1.8 m deep × 0.9 m wide) in two rooms, with 6 birds in each room (3 males and 3 females) (Supplementary Figure S2). Outside of training and testing, magpies from the same room could see and hear one another. Each aviary contained two rectangular perches (15 cm wide), one 1.3 m above the floor at the back of the aviary and the other 0.45 m above the floor at the front of the aviary; aviaries also contained a dowel perch 0.45 m above the floor at the front. Additionally, all aviaries were equipped with two infrared video cameras; one positioned at the highest perch where the magpies usually slept, and the other was mounted on the aviary door focussing on the lower perches and the aviary floor. Both rooms were temperature controlled (22 ± 5 C) and insulated from all external light. Room lighting (153 ± 18 lx) was kept on a 12:12 light:dark cycle (lights-off at 1800 h). A light imitating the intensity of the full moon (average ~ 0.1 lx at the level of the highest perch) was present in each room, allowing each magpie to move safely in their aviary at night.

The birds were fed at 0900 ± 1 h a daily diet of 55 g minced meat mixed with an insectivore mix (Wombaroo Food Products, Australia) and added calcium powder. Magpies had access to clean water ad libitum in a large bowl, allowing the birds to both drink and bathe. Water was changed at least daily. Aviary floors were covered in woodchips. To provide enrichment, 15 – 20 mealworms were scattered on the woodchip-covered floor daily giving the magpies an opportunity to forage on the ground, as they do in the wild. Magpies were habituated to laboratory conditions for *ca.* five months before commencing these experiments.

### EEG and EMG electrode implantation and recordings

To confirm the effectiveness of our sleep deprivation and to quantify changes in sleep architecture, we surgically implanted electrodes over the surface of the brain to record the electroencephalogram, and over the neck muscle for an electromyogram (for details see^60^). Once electrodes were in position, electrode wires were consolidated to form a head plug using dental acrylic. Wires terminated at a connector to which a data logger (Neurologger 2A) would later connect to record the neurophysiological data. In addition to recording brain activity and muscle tone, the data logger had a 3-dimensional accelerometer for capturing head movements. Electrode implantations were done *ca*. three months prior to experiments commencing.

### Experimental design

**Figure 1:**
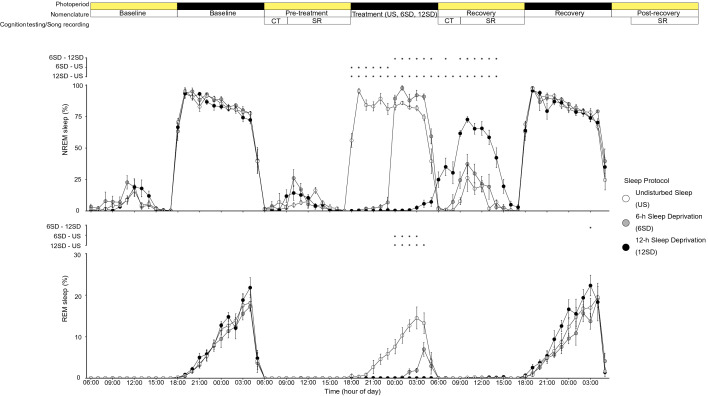
Temporal distribution of NREM and REM sleep of Australian magpies over 72-h EEG recordings for the undisturbed sleep (US), 6-h sleep deprivation (6SD), and 12-h sleep deprivation (12SD) protocols; values are hourly means (%) ± standard error. Significant differences between sleep protocols are shown by asterisks (*lmer* model and Tukey post hoc test, *p* < 0.05). The horizontal bars along the top of the figure indicate the photoperiod with daytime in yellow and nighttime in black, and the corresponding nomenclature for each day/night of the experiments, and whether cognitive testing (CT) or song recording (SR) took place.

Australian magpies were placed in three different sleep protocols/treatments: undisturbed sleep (US; sample size *n* = 9), 6-h sleep deprivation (6SD; *n* = 6), and 12-h sleep deprivation (12SD; *n* = 8) (Fig. 1). Individual birds were used in multiple treatments; order effects were minimized (but not eliminated) by counter-balancing the order of the first two treatments (Supplementary Table S1). Magpies were kept awake by approaching or tapping the aviary, making a noise, or towards the end of the night, gently touching the birds whenever they appeared restful^60^. In the US protocol, the birds were allowed to sleep undisturbed for the entire night; in the 6SD protocol, the birds were deprived of sleep for the first one-half of the night (1800–0000 h); in the 12SD protocol, the birds were kept awake for the entire night (1800–0600 h). The US group served as the control for the reversal learning test. Each protocol consisted of 24-h of habituation to an EEG/EMG data logger, 24-h of baseline sleep and a 12-h pre-treatment day, then a 12-h treatment night (either US, 6SD or 12SD) and 36-h recovery (note, ‘recovery’ simply refers to the time after sleep deprivation). Electrophysiological data were recorded throughout the protocols, except for the habituation period and the post-recovery day. The morning before each treatment night (0600–0900 h) all magpies were tested, and met the success criterion, on a colour association task for which they had been trained two days before testing. The success criterion for associative (and reversal) learning tasks was adopted from Ashton et al.^56^ and defined as making the correct choice in at least 10 of 12 consecutive trials (binomial test: *p* = 0.039). The morning after each treatment (0600–0900 h) the magpies were tested on a reversal learning task. For the 12SD protocol only, we also recorded songs for 3 days (during the pre-treatment, recovery, and post-recovery days); the pre-treatment day acted as a baseline recording between 0900 and 1800 h.

### Cognitive testing apparatus

**Figure 2:**
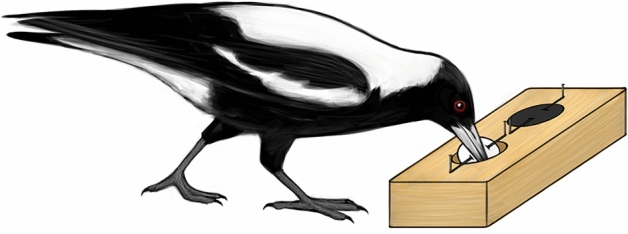
Cognitive testing apparatus. An Australian magpie performs the associative or reversal learning task. Illustration by Laura X. Tan.

To test associative learning, we used an achromatic colour-discrimination task consisting of a wooden foraging grid (18.5 cm long × 9 cm wide × 4.5 cm high) containing two wells (2 cm deep, 3.3 cm diameter). Each well was covered with an opaque acrylic lid that fitted exactly into the wells and were held in place by elastic bands threaded through drilled holes in the lids and fastened to either side of the well. This created an axis on which the lids could swivel when pecked^56^ (Fig. 2).

### Initial training

We trained the magpies to gain access to a food reward (a small amount of grated cheese and mealworms) by first exposing the magpies to the wells without any lids covering them; second with the lids partially covering the wells; and third with the lids fully covering the wells. Lid colour in the training phase was grey, an achromatic colour not used in any of the experimental trials.

Two days before being tested on the associative learning task, each bird was presented with the testing apparatus. Here, the first colour that was chosen (either black or white) became the rewarded colour in the associative learning task; half of the birds chose to begin with black and half chose white (Supplementary Table S1). Each magpie was then given a maximum of 15 training trials on the associative learning task. This training procedure was done to make sure all birds passed the task on the morning after baseline night, so they could all undergo the sleep deprivation on the same night.

### Associative and reversal learning

On the morning after baseline night (0600–0900 h) the magpies were tested on an associative learning task. During experimental trials, one well was covered by a black lid and the other well by a white lid. A bird could search both wells in the first trial to demonstrate that only one of the wells contained a food reward (chilled, unmoving mealworms). In all subsequent trials, each bird was only allowed to search one well before the testing apparatus was removed. To eliminate side biases and to ensure that the colour was the cue being associated with a food reward, the position of the baited well was pseudo-randomized and was never on the same side of the foraging grid for more than two consecutive trials. Furthermore, both wells had previously contained mealworms to control for olfactory cues. An individual was considered to have succeeded at the task when it pecked the rewarded stimulus in at least 10 out of 12 successive trials (binomial test: *p* = 0.039). The number of trials taken to reach this success criterion (including the final 12 trials) was the associative learning score. During testing, magpies were visually separated from each other by a curtain (a plastic tarpaulin) to eliminate the possibility of social learning.

Twenty-four hours after completing the associative learning task, individuals were tested on a reversal learning task until they either reached the success criterion or failed to do so after three hours. The reversal learning task commenced at 0600 h and continued no later than 0900 h on the recovery day (i.e. the day after experimental treatment). The same foraging grid was presented; the only difference being the achromatic colour of the rewarded lid was reversed from that of the associative learning task. Otherwise, the experimental protocol and the success criterion were the same as for the associative learning task described above. Ultimately, we measured (1) latency to first choice (a slower response could reflect reduced attention and/or motivation), (2) number of attempts until success criterion was reached, and (3) the fraction of correct choices; the latter two measurements reflect behavioural flexibility. We also measured (4) whether birds passed (i.e. met criterion) or failed as a dichotomous variable. We tried to test the same individuals in all treatments (US, 6SD, and 12SD); half of the birds started with US, and the other half with 12SD. Owing to reduced task engagement after 12SD, we added a 6SD treatment. Thus, all birds that underwent 6SD had already been through both US and 12SD. Birds were given 1–3 weeks of down-time before going through another treatment.

### Song recording and analysis

During pre-treatment, recovery, and post-recovery days of the 12SD protocol, we opted to record the birds’ songs ad hoc in the morning (0900–1200 h; note, the start time of 0900 h since reversal learning testing occurred 0600–0900), afternoon (1200–1500 h) and evening (1500–1800 h). To record the songs, we used a Zoom H6 digital recorder (Hauppauge, NY, USA) with two Sennheiser K6 microphones (Sennheiser electronic GmbH&Co. KG, USA) facing the aviaries. All birds within the same room were measured together, because isolating social birds could cause stress and confound results. From the recordings, we manually selected all singing bouts longer than 0.3 s and a song bout was considered separate from another bout only if there was at least two seconds of silence between them. We measured the total number, duration, and timing of songs, and minimum and maximum frequencies (kHz) using Raven Pro 1.5 (Cornell Lab of Ornithology, NY, USA).

### Sleep staging

The electrophysiological sleep data presented here are a subset of data published elsewhere^60^, with the addition here of a control undisturbed sleep protocol (US), which we opted to omit from the article published previously. We used the supervised machine-learning algorithm Somnivore™^61^ to score wakefulness, NREM sleep, and REM sleep in 4-s epochs. Somnivore has been validated for use on Australian magpies^62^ and other animals^61^.

### Statistics

We tried to use each bird in each experimental treatment. However, of the 12 magpies, we ultimately tested 8 (reversal learning: 12SD), 6 (reversal learning: 6SD), 9 (reversal learning: US), and 11 (song: 12SD) birds (Supplementary Table S1). Discrepancies in sample sizes arose from (1) not testing the birds without EEG/EMG electrodes on reversal learning, and (2) unexpected data logger failures or loggers detaching from birds.

The effects of extended wakefulness on subsequent sleep was analyzed using the *lmer* model and Tukey post hoc tests (but see Johnsson et al. for in-depth analysis of sleep)^60^. For the reversal learning task, we used a one-way ANOVA followed by post hoc Welch two sample t-tests when comparing latency to first choice (reflects motivation and/or attention), and number of attempts until success criterion was reached and fraction of correct choices (reflects behavioural flexibility) between the different sleep deprivation treatments. All latency values were log10 transformed to fit assumptions of normality. We also analysed whether pass-or-fail on the reversal learning task differed between treatments using a binary logistic regression. Generalized linear models (GLM) with post hoc Benjamin-Hochberg corrections were used to compare songs between the pre-treatment day (control), recovery day, and post-recovery day. For the GLM we used two response variables: song duration and number of songs. Since we could not separate birds and because they sing together, we considered the birds as a group. Most statistical analyses were conducted in R version 3.5.2 (R Development Core Team 2018); the logistic regression was done in Systat 13.

### Ethics and permissions

After the study, the magpies were released back into the wild in July 2019. All methods were approved by the La Trobe University Animal Ethics Committee (AEC 18034), the Department of Environment, Land, Water and Planning (permit number: 10008264), and the Australian Bird and Bat Banding Scheme (ABBBS number 1405). The authors confirm that all experiments were performed in accordance with relevant guidelines and regulations.

## Results

The effects of extending wakefulness on subsequent sleep amount, timing, composition, continuity, and intensity were many and diverse, yet the specific details are not relevant here. Instead, we direct those interested to our previously published article for a thorough discussion of sleep homeostasis in Australian magpies^60^. What is important for our present purpose is that we were able to virtually eliminate NREM and REM sleep during both 6SD and 12SD (Fig. 1). Following all-night sleep loss, magpies recovered lost NREM sleep by sleeping during the day; after sleep deprivation during the first-half of the night, the birds recovered lost NREM sleep by engaging in more, and more intense, NREM sleep. Lost REM sleep was not recovered. By the recovery night, sleep amount and composition had returned to baseline levels. Next, we detail our findings related to attention and/or motivation (latency to make the first choice), behavioural flexibility (number of attempts until success criterion was reached; fraction of correct choices), and song output (number, duration, and frequency bandwidth of songs).

### Associative and reversal learning

All birds that were tested on the associative learning task passed the success criterion and were allowed to proceed onto the reversal learning task the following day. The average number of trials on the associative learning task was 12.9 ± 1.5 (before US), 11.7 ± 0.6 (before 6SD), and 16.4 ± 3.4 (before 12SD) (Supplementary Table S2).

**Figure 3:**
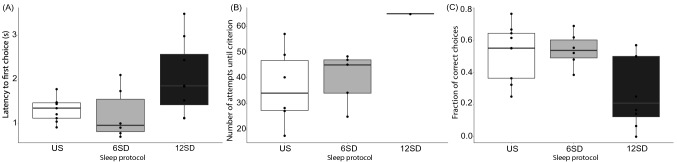
Cognitive testing scores on the reversal learning task for Australian magpies in the undisturbed sleep (US), 6-h sleep deprivation (6SD), and 12-h sleep deprivation (12SD) protocols showing the latency to first choice (**A**), the number of attempts needed to reach the success criterion (**B**), and the fraction of correct choices (**C**).

On the reversal learning task, the time taken for birds to make their first choice increased during 12SD compared to US (*t* = 2.322; d*f* = 8.21; *p* = 0.048) and to 6SD (*t* = 2.191; d*f* = 11.90; p = 0.049); this latency was not different between US and 6SD (*t* = − 0.420; d*f* = 6.47; *p* = 0.688) (Fig. 3A; Supplementary Table S2). There was no difference of performance (measured as the number of attempts until success criterion was reached) between US and 6SD (*t* = 0.457; d*f* = 8.66; *p* = 0.659) (Fig. 3B). After 12SD, only 1 of 8 magpies succeeded on completing the task (i.e. only one reached success criterion during the three hours allotted), which was not amenable to statistical analysis. However, the one bird that succeeded nevertheless required the highest number of attempts to succeed. The fraction of correct choices made during the reversal learning task was lower after 12SD compared to both 6SD and US (*F* = 5.427; d*f* = 2; *p* = 0.013) (*t* = − 2.999; d*f* = 10.61; *p* = 0.013; and *t* = − 2.586; d*f* = 13.38; *p* = 0.022, respectively); there was no difference between 6SD and US (*t* = − 0.218 ; d*f* = 12.96; *p* = 0.831) (Fig. 3C). We also found that the ability to succeed on the reversal learning task depended on how many hours the birds been kept awake (pass-or-fail logistic regression: *z* = 2.110; *p* = 0.035).

### Song output

**Figure 4:**
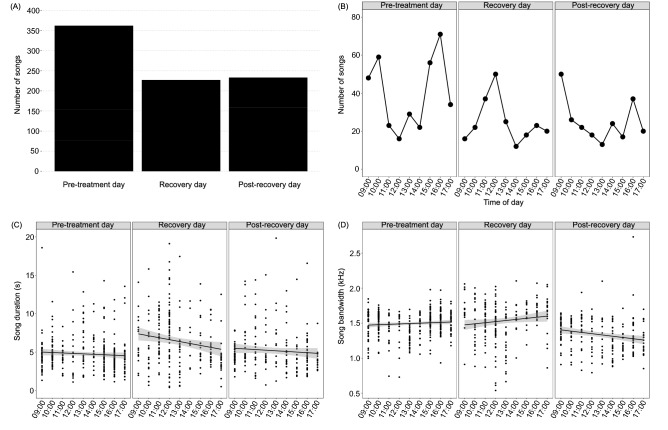
Effect of 12-h sleep deprivation (12SD) on the songs of Australian magpies. The number (**A**), timing (**B**), duration (**C**), and frequency bandwidth (**D**) of songs per day.

After all-night sleep loss (12SD), Australian magpies sang less (*z* = − 5.750; *p* < 0.001), and continued singing less during the post-recovery day (*z* = − 5.169; *p* < 0.001) (Fig. 4A). Magpies also changed the timing of their singing. During baseline conditions, magpies sang predominantly in the morning and evening, which is typical for this species in the wild^55^. However, after sleep deprivation, they sang mostly mid-day (Fig. 4B). Interestingly, the timing of songs reverted to morning and evening during the post-recovery day, even while the birds still sang less overall. Despite singing approximately one-third less after a night of sleep loss, songs were longer after sleep deprivation (*t* = 6.573; d*f* = 805; *p* < 0.001), before returning to baseline levels the following day (Fig. 4C). In addition to changes in the amount, timing and duration of singing, the frequency bandwidth of songs narrowed during the post-recovery day (*t* = -7.892; d*f* = 805; *p* < 0.001) (Fig. 4D), caused by both an increase in the minimum frequency (*t* = 5.434; d*f* = 805; *p* < 0.001) and a decrease in the maximum frequency of songs (*t* = − 6.905; d*f* = 805; *p* < 0.001).

## Discussion

Our results on Australian magpies are in accordance with previous research on humans in that sleep deprivation impairs cognition and alters communication^8,14,63,64^. We found that magpies, a bird with advanced cognitive abilities^56^, kept awake for an entire night have impaired motivation, attention, and behavioural flexibility. Moreover, our impromptu recordings of songs hinted that these social birds changed their song timing and output. Thus, many aspects of cognition appear to be sleep-dependent in Australian magpies.

Australian magpies were more likely to fail at the reversal learning task after a full night of sleep deprivation compared to half-night sleep deprivation and undisturbed sleep. Similarly, magpies made fewer correct choices and took longer to make their first choice when awake longer. For performance on the reversal learning task, only one magpie reached success criterion after 12SD, as tired magpies lost motivation and/or prioritized daytime sleep rather than interacting with the test. The similarity of our measures of cognitive performance on the reversal learning task between 6SD and US is perhaps not surprising given magpies had recovered lost sleep during the last half of the treatment night^60^. In humans, insufficient sleep has detrimental effects on cognitive performance, and cognitive measures such as motivation, alertness, attention, and vigilance are especially affected by sleep loss^14,65,66^. These parameters may serve as a foundation for more advanced cognition since cognitive processes rely on alertness and attention^14^.

Tired magpies are inattentive and unmotivated. However, from these data, we cannot determine which aspect of cognition was most affected by sleep loss. To do so, we would need to design a study with multiple tests, each targeting one specific measure of cognition. For example, the psychomotor vigilance test measures reaction time and is commonly used in human sleep deprivation studies to evaluate alertness and vigilance^67^. If repurposed for birds, we could evaluate the relative vulnerability of alertness and vigilance by measuring reaction time and see how the ability to sustain vigilance changes over the duration of the task. This time-on-task effect is the progressive worsening of performance with sustained focus on a cognitive task and has been shown to be greatly affected by sleep deprivation^14,65,68^. In turn, the time-on-task effect seems to be strongly affected by motivation^69^, which might explain why our magpies performed worse after 12 h of sleep deprivation. That said, it is more likely that sleepiness itself might have reduced motivation in the magpies, as has been shown in sleep deprived humans that become less willing to engage in physical activities after insufficient sleep^70^. Therefore, a lack of motivation is likely to have caused a decrease in performance on the reversal learning task and altered singing in our magpies. Since sleep deprivation can affect many different cognitive processes, it is important for future studies to consider specific task requirements when interpreting measures of cognitive performance^71^.

In our study, we found that behavioural flexibility, as measured by reversal learning, was impaired by sleep deprivation. These results are comparable to studies done on other animals where sleep deprivation has been shown to have negative impacts on different aspects of cognition, such as long-term memory and foraging efficiency in lemurs^7^, and object discrimination and avoidance learning in zebrafish (*Danio rerio*)^4,5^. Dose-dependent effects of extending wakefulness have been explored in other taxa, but with inconsistent results. In the study on zebrafish avoidance learning, partially sleep deprived zebrafish (extended light phase from 12 to 16 h) performed similar to undisturbed fish^5^, perhaps, as with our magpies, owing to sleep rebound during the remainder of the night^60^. In contrast, in humans, chronic sleep restriction caused a compounding and dose-dependent deficit in cognitive performance^12^. Our study would have benefited from an additional 6SD group, one sleep deprived during the last half of the night to prevent recovery sleep prior to testing. Nevertheless, the present study shows that sleep deprivation can cause deficits in behavioural flexibility on a reversal learning task in birds.

Song performance in Australian magpies was affected by sleep loss. Tired magpies sang less, an effect that persisted even after a full night of recovery sleep. Surprisingly, however, when the magpies did sing, they sang longer. On the post-recovery day, songs had a narrower frequency bandwidth, an effect that was not present on the day immediately following sleep loss. It remains unclear whether the alteration of song is an impairment or adaptive, or whether these alterations influence the perception of the song from the perspective of the receiver.

Australian magpies produce complex vocalizations that are used in the context of territory defense and social bonding within the family group^57^. Perhaps successful defense and bonding may require that magpies are able to recognize competing neighbours and family members based on their songs and other vocalizations^47^. Individual fitness may be negatively affected by a distortion of the information content of the signal. Indeed, other species of songbirds that live in urban habitats shift their song frequencies toward higher frequencies when exposed to interfering anthropogenic noise^72,73^. In a study by Halfwerk and colleagues on singing in great tits, urban noise impairs male–female communication, and signal efficiency depended on song frequency, revealing the importance of the frequency component of songs in communication (at least in the presence of anthropogenic noise)^74^. So, while we know that even modest changes in song structure can have an effect on communication among songbirds, whether alteration in song frequencies caused by sleep deprivation has an effect on communication, as a whole, remains unclear.

Communication in some animals is affected by sleep loss. The ability of honeybees (*Apis mellifera*) to communicate the direction and distance to a food resource via their waggle dance is impaired following nighttime sleep loss^75^. Human communication is also impaired by sleep loss, including slower, softer, slurred, and mumbled speech to the point where the listener cannot understand what the person speaking is saying^76^. The content of speech after an extended period of wakefulness shows an increase in vagueness, repetitions, abrupt shifts in topic, unfinished sentences, mispronunciations, omission of syllables, and fusion of words; all of which are accompanied by less self-correction of speech errors as wakefulness progresses^76^. In addition, extended wakefulness results in a more variable fundamental frequency and word duration^77^, deterioration in word generation, and a reduction in appropriate intonation with more monotonic voices^63^, impaired verbal working memory^78^, and reduced performance on complex language tasks^79,80^. Thus, communication in birds, bees, and humans is altered with extended periods of wakefulness.

A caveat with our study is that we could not fully isolate the birds. The magpies could see and hear one another and likely influenced one another to sing. Since singing is often done in groups of birds in the wild, if one bird started singing in one of our experimental rooms, then this might have increased the likelihood of other birds singing^55^; that said, the non-independence of singing was at least consistent across all days. Also, we were unable to identify which individuals were singing and for how long, such that some birds may have been singing more than others. With this caveat in mind, it is important to note that this aspect of the study was unplanned and therefore birds could not be fully isolated. That said, magpies are social in nature, and complete isolation may have caused ‘unnatural’ behavioural responses arising from such an acute change to their social environment. Nonetheless, this study provides a first window into sleep-dependent changes in singing in adult songbirds and suggests further research is needed.

### Conclusion

Sleep deprived Australian magpies were slower to attempt the reversal learning task, less likely to perform and complete the task, and the individual that completed the task performed worse than better-rested birds. After extended wakefulness magpies seemed to prioritize sleep over singing, but songs were longer, shifted towards mid-day, and with a narrower bandwidth the following day. However, future studies should verify our song-related results. Overall, these findings indicate that sleep affects ecologically-relevant behaviours, including behavioural flexibility and singing in adult songbirds.

## Supplementary Information

Supplementary Information 1.

Supplementary Information 2.

Supplementary Information 3.

Supplementary Information 4.

Supplementary Information 5.

Supplementary Information 6.
