# Supplementary figures and images for: Sleep loss impairs cognitive performance and alters song output in Australian magpies
Source: Sci Rep. 2022 Apr 22;12:6645. doi: 10.1038/s41598-022-10162-7 (PMC9033856; doi:10.1038/s41598-022-10162-7)

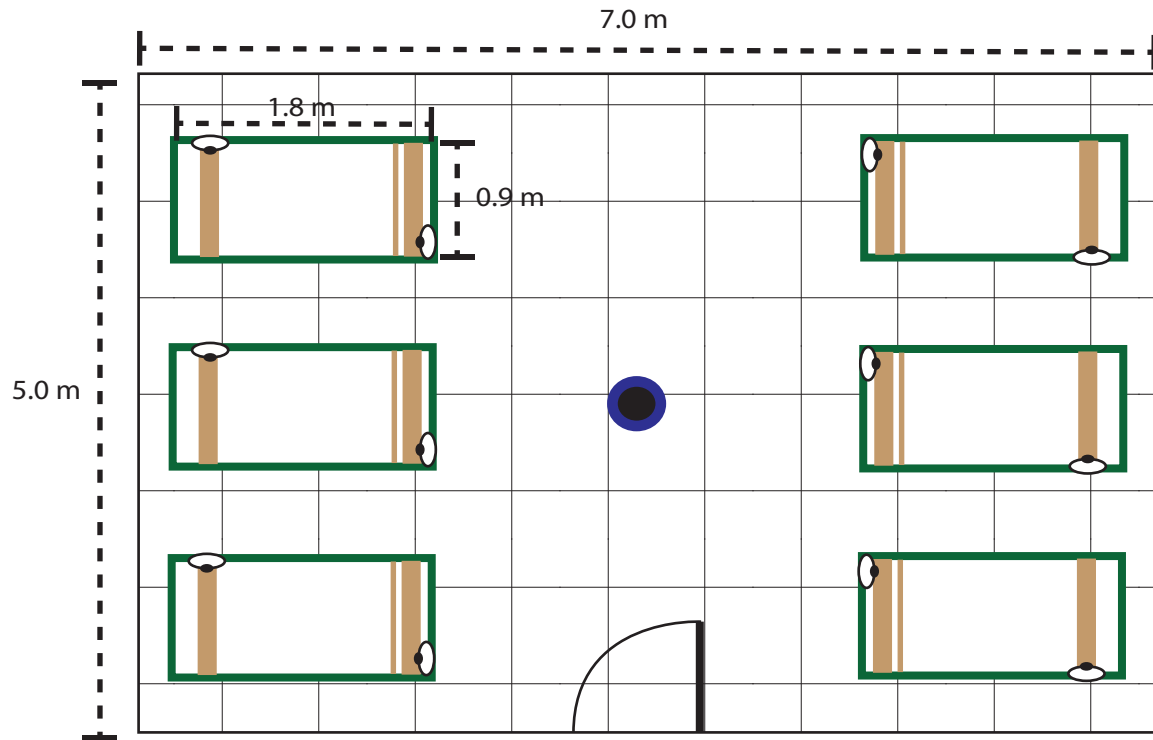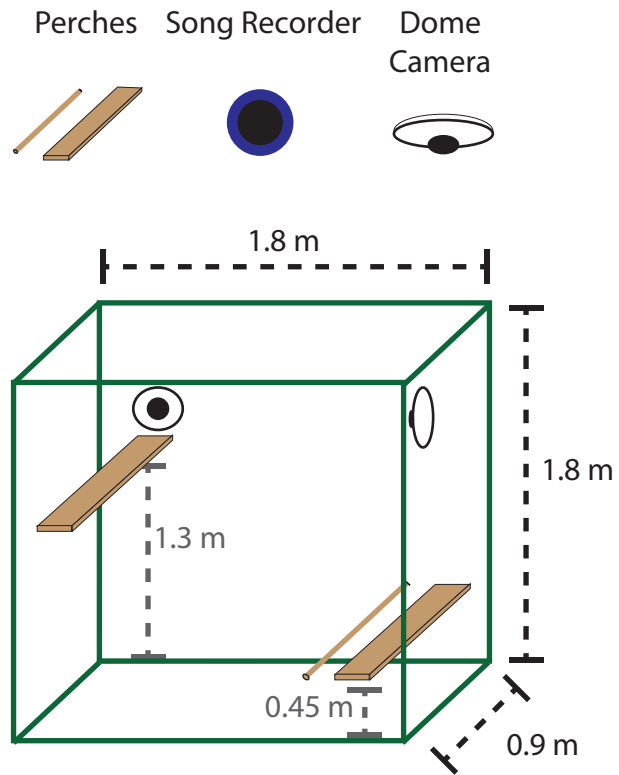

Supplement: Supplementary file 2 — Supplementary Information 2. [file 41598_2022_10162_MOESM2_ESM.pdf]

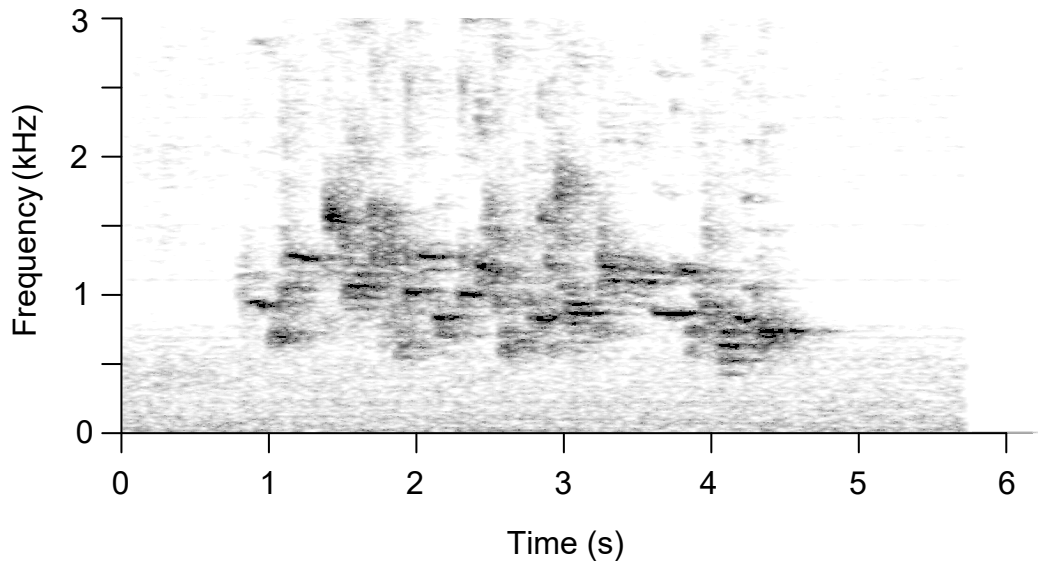

Supplement: Supplementary file 3 — Supplementary Information 3. [file 41598_2022_10162_MOESM3_ESM.pdf]
