# Supplementary material for: Sleep loss impairs cognitive performance and alters song output in Australian magpies
Source: Sci Rep. 2022 Apr 22;12:6645. doi: 10.1038/s41598-022-10162-7 (PMC9033856; doi:10.1038/s41598-022-10162-7)
Supplement: Supplementary file 4 — Supplementary Information 4. [file 41598_2022_10162_MOESM4_ESM.docx]

**Supplementary Table S1.** Details of cognitive testing. Nine of twelve birds (ID) were tested on the reversal learning task for each sleep protocol (undisturbed sleep [US]; 6-h sleep deprivation [6SD]; 12-h sleep deprivation [12SD]). The starting colour chosen by each bird, and the protocol order, are also provided.

|  | Tested or not tested | | | Starting colour | | | Protocol order | | |
| --- | --- | --- | --- | --- | --- | --- | --- | --- | --- |
| ID | US | 6SD | 12SD | US | 6SD | 12SD | First | Second | Third |
| Taylor | tested | not tested | not tested | Black | . | . | US | . | . |
| Darcy | tested | tested | tested | Black | Black | Black | 12SD | US | 6SD |
| Varcoe | tested | tested | tested | White | White | White | 12SD | US | 6SD |
| Goldsack | tested | not tested | tested | Black | . | Black | 12SD | US | . |
| Sidebottom | not tested | not tested | not tested | . | . | . | . | . | . |
| Grundy | tested | tested | tested | White | White | White | 12SD | US | 6SD |
| Swan | tested | tested | tested | Black | White | Black | US | 12SD | 6SD |
| Pendlebury | not tested | not tested | not tested | . | . | . | . | . | . |
| Tufty | not tested | not tested | not tested | . | . | . | . | . | . |
| DeGoey | tested | tested | tested | Black | White | White | US | 12SD | 6SD |
| Cox | tested | not tested | tested | White | . | White | US | 12SD | . |
| Treloar | tested | tested | tested | White | Black | White | US | 12SD | 6SD |
