# Supplementary material for: Sleep loss impairs cognitive performance and alters song output in Australian magpies
Source: Sci Rep. 2022 Apr 22;12:6645. doi: 10.1038/s41598-022-10162-7 (PMC9033856; doi:10.1038/s41598-022-10162-7)
Supplement: Supplementary file 5 — Supplementary Information 5. [file 41598_2022_10162_MOESM5_ESM.docx]

**Supplementary Table S2.** Raw data for the birds (ID) tested in each sleep protocol (undisturbed sleep [US]; 6-h sleep deprivation [6SD]; 12-h sleep deprivation [12SD]). For the colour association task (CA), the table shows number of attempts each bird did on the task before each treatment. For the reversal learning task (RL), the table shows latency to make a first choice (in seconds), number of attempts, number of attempts until reaching success criterion (10 out of 12 correct choices within three hours), the fraction of correct choices on the task, and whether each bird passed or failed the task.

| ID | Sleep  protocol | CA attempts until criterion | RL latency to first choice | RL number of attempts | RL attempts until criterion | RL fraction of correct choices | RL  pass-or-fail |
| --- | --- | --- | --- | --- | --- | --- | --- |
| Taylor | US | 12 | 16 | 28 | 28 | 0.643 | pass |
| Darcy | US | 10 | 22 | 12 | . | 0.250 | fail |
| Varcoe | US | 12 | 60 | 27 | 27 | 0.667 | pass |
| Goldsack | US | 10 | 30 | 11 | . | 0.364 | fail |
| Grundy | US | 25 | 27 | 17 | 17 | 0.765 | pass |
| Swan | US | 11 | 13 | 57 | 57 | 0.614 | pass |
| DeGoey | US | 13 | 8 | 49 | 49 | 0.551 | pass |
| Cox | US | 12 | 29 | 31 | . | 0.323 | fail |
| Treloar | US | 11 | 11 | 40 | 40 | 0.550 | pass |
| Darcy | 6SD | 12 | 53 | 26 | . | 0.385 | fail |
| Varcoe | 6SD | 13 | 126 | 25 | 25 | 0.480 | pass |
| Grundy | 6SD | 13 | 6 | 48 | 48 | 0.521 | pass |
| Swan | 6SD | 10 | 8 | 34 | 34 | 0.618 | pass |
| DeGoey | 6SD | 12 | 5 | 47 | 47 | 0.553 | pass |
| Treloar | 6SD | 10 | 10 | 45 | 45 | 0.689 | pass |
| Darcy | 12SD | 10 | 33 | 8 | . | 0.250 | fail |
| Varcoe | 12SD | 32 | 69 | 2 | . | 0.500 | fail |
| Goldsack | 12SD | 12 | . | 0 | . | 0.000 | fail |
| Grundy | 12SD | 12 | 72 | 24 | . | 0.500 | fail |
| Swan | 12SD | 12 | 13 | 65 | 65 | 0.569 | pass |
| DeGoey | 12SD | 10 | 13 | 15 | . | 0.067 | fail |
| Cox | 12SD | 32 | 269 | 6 | . | 0.167 | fail |
| Treloar | 12SD | 11 | 934 | 7 | . | 0.143 | fail |
