# Supplementary material for: Sleep loss impairs cognitive performance and alters song output in Australian magpies
Source: Sci Rep. 2022 Apr 22;12:6645. doi: 10.1038/s41598-022-10162-7 (PMC9033856; doi:10.1038/s41598-022-10162-7)
Supplement: Supplementary file 6 — Supplementary Information 6. [file 41598_2022_10162_MOESM6_ESM.docx]

**Supplementary legends**

**Figure S1.** Experimental room. Note that one of the two rooms housed only five magpies. Adapted with permission from Connelly et al. (2020).

**Figure S2.** Spectrogram of a single Australian magpie singing. Produced in R, using the package *soundgen*. Parameters: bit = 16, sampling rate: 44100 Hz, FFT window length = 120 ms, window type = hanning, overlap = 90%, noise reduction: 0.4.

**Audio S1.** Example of an Australian magpie singing.
